# Enzymes Involved in the Biosynthesis of Arginine from Ornithine in Maritime Pine (*Pinus pinaster* Ait.)

**DOI:** 10.3390/plants9101271

**Published:** 2020-09-27

**Authors:** José Alberto Urbano-Gámez, Jorge El-Azaz, Concepción Ávila, Fernando N. de la Torre, Francisco M. Cánovas

**Affiliations:** Grupo de Biología Molecular y Biotecnología, Departamento de Biología Molecular y Bioquímica, Universidad de Málaga, Campus Universitario de Teatinos, 29071 Málaga, Spain; alburb@uma.es (J.A.U.-G.); jelazaz@alu.uma.es (J.E.-A.); cavila@uma.es (C.Á.)

**Keywords:** arginine, ornithine, *Pinus*, conifers, nitrogen metabolism, amino acid biosynthesis, enzyme kinetics, biochemical regulation

## Abstract

The amino acids arginine and ornithine are the precursors of a wide range of nitrogenous compounds in all living organisms. The metabolic conversion of ornithine into arginine is catalyzed by the sequential activities of the enzymes ornithine transcarbamylase (OTC), argininosuccinate synthetase (ASSY) and argininosuccinate lyase (ASL). Because of their roles in the urea cycle, these enzymes have been purified and extensively studied in a variety of animal models. However, the available information about their molecular characteristics, kinetic and regulatory properties is relatively limited in plants. In conifers, arginine plays a crucial role as a main constituent of N-rich storage proteins in seeds and serves as the main source of nitrogen for the germinating embryo. In this work, recombinant PpOTC, PpASSY and PpASL enzymes from maritime pine (*Pinus pinaster* Ait.) were produced in *Escherichia coli* to enable study of their molecular and kinetics properties. The results reported here provide a molecular basis for the regulation of arginine and ornithine metabolism at the enzymatic level, suggesting that the reaction catalyzed by OTC is a regulatory target in the homeostasis of ornithine pools that can be either used for the biosynthesis of arginine in plastids or other nitrogenous compounds in the cytosol.

## 1. Introduction

Nitrogen (N) is an essential constituent of proteins, nucleic acids and many other compounds essential for life such as hormones, vitamins, porphyrins and a wide range of specialized metabolites. Although N is highly abundant in the atmosphere as dinitrogen, only combined forms such as nitrate, ammonium or amino acids can be assimilated by plants. Because these forms of N are usually present in low abundance in nature, N availability is a major limiting factor for plant growth and development [1]. When an excess of N is available, plants can assimilate and store it directly as free arginine, the amino acid with the highest N content or as a component of storage proteins in the bark and the seeds [2,3]. Therefore, arginine is of paramount importance in N metabolism, and therefore the study of its biosynthesis and utilization is essential to understand N homeostasis.

The biosynthesis of arginine can be divided into two pathways: the conversion of glutamate into ornithine and the synthesis of arginine from ornithine through the sequential action of the enzymes ornithine transcarbamylase (OTC), argininosuccinate synthetase (ASSY) and argininosuccinate lyase (ASL) [4,5].

**Figure 1:**
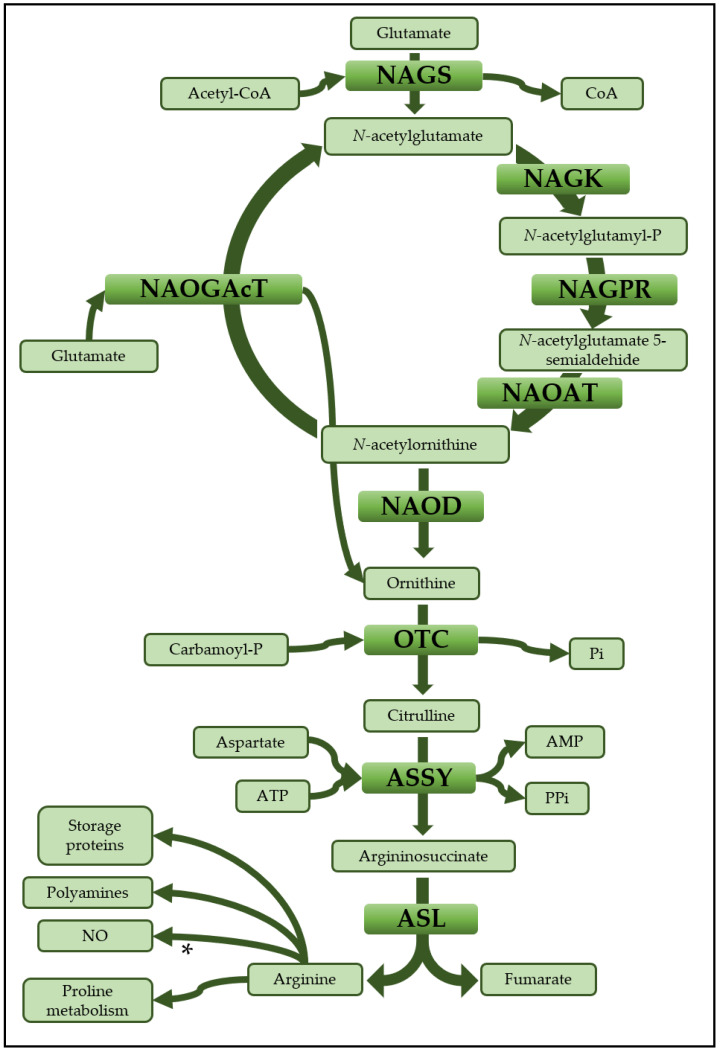
**Arginine biosynthesis**. Schematic pathway of arginine biosynthesis from ornithine with the main acting molecules and possible metabolic destinations for arginine. * Although the implication of arginine in the biosynthesis of NO has been established in animals, it remains a matter of debate in plants. NAGS: *N*-acetylglutamate synthase; NAGK: *N*-acetylglutamate kinase; NAGPR: *N*-acetylglutamyl-phosphate reductase; NAOAT: *N*-acetylornithine aminotransferase; NAOGAcT: *N*-acetylornithine:glutamate acetyltransferase; NAOD: *N*-acetylornithine deacetylase; OTC: Ornithine transcarbamilase; ASSY: Argininosuccinate synthetase; ASL: Argininosuccinate lyase.

Figure 1 shows a schematic representation of the enzymatic steps involved in the conversion ornithine-arginine and the metabolic fates of arginine as a precursor for the biosynthesis of polyamines, storage proteins and nitric oxide (NO). The enzymatic generation of NO from arginine has been demonstrated biochemically, but the identity of the protein and gene responsible for this conversion in plants remain unknown [6]. When the arginine metabolic pathway is compared among different live forms an outstanding characteristic is its metabolic plasticity, enabling it being able to adapt and meet the requirements of various organisms to maintain an appropriate N balance. For example, in the urea cycle in mammals the arginine biosynthetic pathway has been adapted for the disposal of ammonium, a highly toxic product generated through the catabolism of the excess proteins ingested in their diet [7]. The enzymes of the urea cycle are located in the hepatocytes [8], where the catalytic action of the mitochondrial enzymes carbamoyl-P synthetase I and OTC convert ammonium into citrulline which is transported to the cytoplasm for the synthesis of arginine by the sequential action of ASSY and ASL. An additional enzyme of the arginine metabolism, arginase (ARG), is critical for the cyclic nature of urea synthesis, catalyzing the formation of the ornithine and urea that is excreted in urine. The enzymes ASSY and ASL have been immunocytochemically located to the immediate vicinity of mitocondria, close to the outer membrane, further supporting the functionality of the cyclic pathway [9]. Given the metabolic importance of this cycle, the enzymes involved in urea biosynthesis have been isolated from different animal sources, including humans, and their molecular and kinetic properties have been determined (Table S1). Their deficiencies have been found to be related to cancer and several metabolic disorders involving N metabolism and causing hyperammonemia [10,11]. Furthermore, the high nutritional value of arginine as a complement to vitamin deficiency in humans has been determined [12]. In contrast, much less is known about the molecular characteristics, kinetic parameters and regulation of enzymes involved in arginine biosynthesis in plants [5] (Table S1).

The compartmentation of arginine biosynthesis in plants was controversial in early studies, and the cytoplasmic and mitochondrial localization of several enzymes was reported [13], as previously described in animals. However, after the completion of the Arabidopsis genome sequence [14], it was found that genes encoding most arginine biosynthetic enzymes contain a presequence for chloroplast targeting [15]. Recent localization studies have confirmed these genomic data and have provided strong evidence showing that the complete pathway is confined to plastids, including the enzymes of ornithine pathway and those involved in the biosynthesis of arginine from ornithine, OTC, ASSY and ASL [4,16,17]. 

Storage proteins are of key importance for the nutrition of conifer germinating seeds. In maritime pine, arginine is one of the most abundant amino acid residues in the storage proteins representing with glutamine/glutamate the majority of the total amino acid content [18]. Storage proteins mainly accumulate in the megagametophyte but also reside in the embryo during the final stages of seed formation [19]. Fundamental knowledge of pine embryogenesis is critical to develop experimental procedures for the multiplication of genotypes of commercial interest through in vitro culture techniques. In fact, somatic embryogenesis in combination with cryopreservation is currently a major biotechnological tool for vegetative propagation of selected maritime pine varieties [20]. However, previous works have shown a deregulation of the arginine biosynthetic pathway and a lower accumulation of storage proteins during the last steps of somatic embryogenesis [4,19].

Current research efforts are addressed to further understand how arginine biosynthesis is regulated during pine embryogenesis. The availability of genomic resources has greatly facilitated the performance of functional studies in maritime pine [21,22]. To further understand the molecular regulation of arginine biosynthesis, the enzymes involved in the metabolic conversion of ornithine to arginine have been overproduced in *E. coli* as recombinant proteins, and their molecular, kinetic and regulatory properties have been determined. The results reported here provide new insights into the short-term biochemical regulation of the arginine biosynthetic pathway.

## 2. Results

### 2.1. Identification of Conserved Protein Motifs in the Catalytic Mechanism of PpOTC, PpASSY and PpASL

In a previous paper, full-length cDNAs of *PpOTC*, *PpASSY* and *PpASL* genes [4] were cloned, their expression was analyzed and the subcellular localization of the corresponding proteins demonstrated their targeting to plastids [4]. Here, a detailed analysis was conducted to identify of the protein motifs involved in their catalytic mechanism.

**Figure 2:**
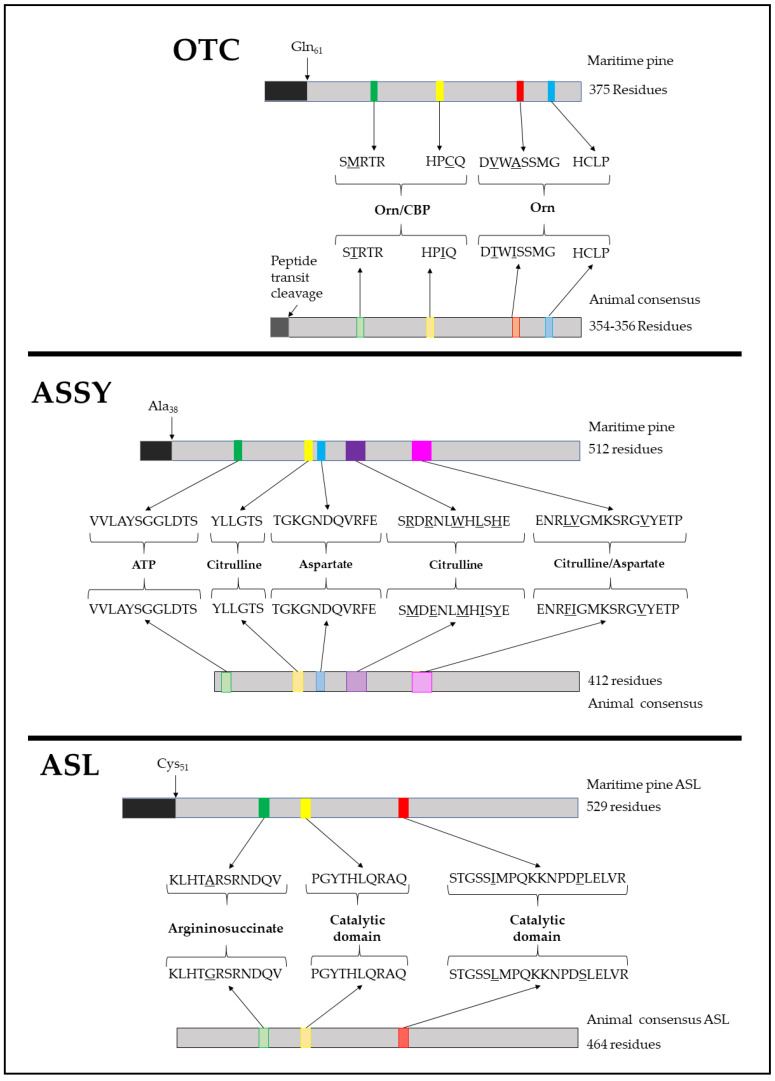
Schematic representation of OTC, ASSY and ASL primary structures. The distribution of conserved protein motifs and the primary structures of the proteins in both plants and animals are shown, with the conserved sequences and the punctual changes in some motifs (underlined). It is also highlighted the predicted cleavage site of the transit peptide using bioinformatics algorithms (TargetP 2.0). “Animal consensus” refers to the integration of sequences from *Homo sapiens*, *Mus musculus*, *Rattus norvegicus*, *Anas platyrhynchos*, *Bos taurus*, *Ovis aries* and *Danio rerio*. Complete alignment for these enzymes is shown in Figure S1.

Through a multiple alignment analysis of PpOTC from different plant and animal species, the characteristics of C-terminal ornithine binding motifs FMHCLP and DVWASMG were identified (consensus DXXXSMG) [23] (Figure 2). The motifs directly involved in the binding of carbamoyl-P, HPCQ and SMRTR, respectively, were also identified (Figure 2, OTC) [23]. For PpASSY, conserved protein motifs involved in the citrulline/aspartate binding loop, ENRLVGMKSRGVYETP, aspartate binding, TGKGNDQVRFE and in the citrulline and ATP binding, SRDRNLWHLSHE [24,25]. Based on sequence homology, the N-terminal domains involved in ATP binding, VVLAYSGGLDTS, and citrulline binding, YLLGTS [26] were also recognized (Figure 2, ASSY). Finally, the PpASL primary structure was also analyzed to detect three characteristic conserved domains involved in the conformation of the catalytic active site: KLHTARSRNDQV, PGYTHLQRAQ and STGSSIMPQKKNPDPMELVR [27] (Figure 2, ASL). The former sequence has also been described as fundamental for argininosuccinate binding [28]. These sequences are highly conserved among the ASL enzymes in plants and animals, as illustrated in Figure S1 and described in the literature [29,30]. Together, these analyses, revealed the critical protein motifs required for the corresponding enzymatic reactions of PpOTC, PpASSY and PpASL.

### 2.2. Production of Recombinant PpOTC, PpASSY and PpASL Enzymes

With the aim of deepening the understanding of the metabolic role of these enzymes, experimental protocols for the production of active recombinant PpOTC, PpASSY and PpASL enzymes in *E. coli* were established to enable the characterization of their molecular and kinetic attributes. For this purpose, the ORFs without the sequence corresponding to the chloroplast transit peptide (CTP) were subcloned into a *pDEST17* vector. The CTPs were removed according to theoretical predictions based on the TargetP-2.0 server [31] and WoLF *PSORT* tools [32]. The proteins were produced as fusions to 6xHis N-terminal tags to confirm their production by western blotting and to facilitate their subsequent purification. Several bacterial strains were used, and different culture conditions were assayed to successfully overexpress the maritime pine enzymes. Figure S2 depicts the expression conditions as optimized for each protein. To confirm that all recombinant polypeptides were correctly expressed, we determined their molecular weights through SDS-PAGE analysis, confirming the predictions based on the sequence analysis: 38 kDa for PpOTC, 47 kDa for PpASSy and 54 kDa for PpASL. The sizes of the individual polypeptides were confirmed with purified preparations of the recombinant enzymes (Figure S3). As shown below, the resulting recombinant purified fractions of PpOTC, PpASSY and PpASL were fully active.

### 2.3. Molecular Size of Maritime Pine OTC, ASSY and ASL Enzymes

**Figure 3:**
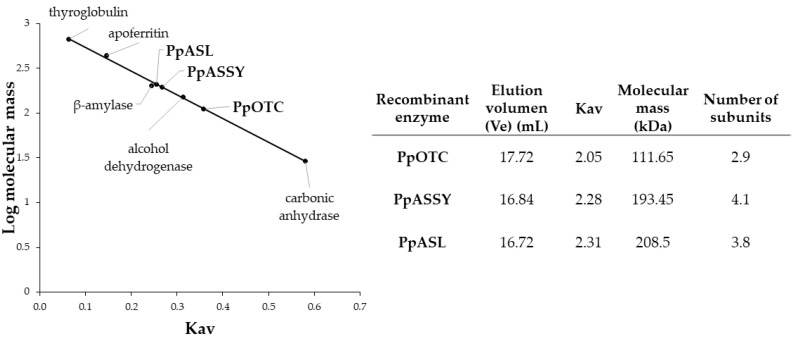
Molecular mass estimations of PpOTC, PpASSY and PpASL. Samples from purified proteins were loaded separately onto a size exclusion ENrich™ SEC 650 column (Bio-Rad). Molecular masses were calculated by comparing the partition coefficient (Kav) of the samples with the standards used to calibrate the column: thyroglobulin (669 kDa), apoferritin (443 kDa), *β*-amylase (200 kDa), alcohol dehydrogenase (150 kDa) and carbonic anhydrase (29 kDa).

Purified preparations of recombinant PpOTC, PpASSY and PpASL enzymes were used to estimate the molecular mass of holoenzymes by gel filtration chromatography through a calibrated column with protein markers of a known size (Figure 3). The recombinant PpOTC holoenzyme showed a size of 112 kDa, 2.9-fold larger than that of the polypeptide and thus consistent with a trimeric structure comprising identical subunits of 38 kDa. The size of the PpASSY holoenzyme was estimated to be 193 kDa, 4.1-fold the mass described for the 47 kDa polypeptide, strongly suggesting a homotetrameric structure. Finally, the size of the PpASL holoenzyme was determined to be 208 kDa, consistent with a homotetrameric structure comprising integrated subunits of 54 kDa. These results are consistent with previous reports about the quaternary structure of these enzymes in bacteria, plants and animals [5,15,33]. 

### 2.4. Catalytic Properties of the Recombinant PpOTC, PpASSY and PpASL Enzymes 

**Figure 4:**
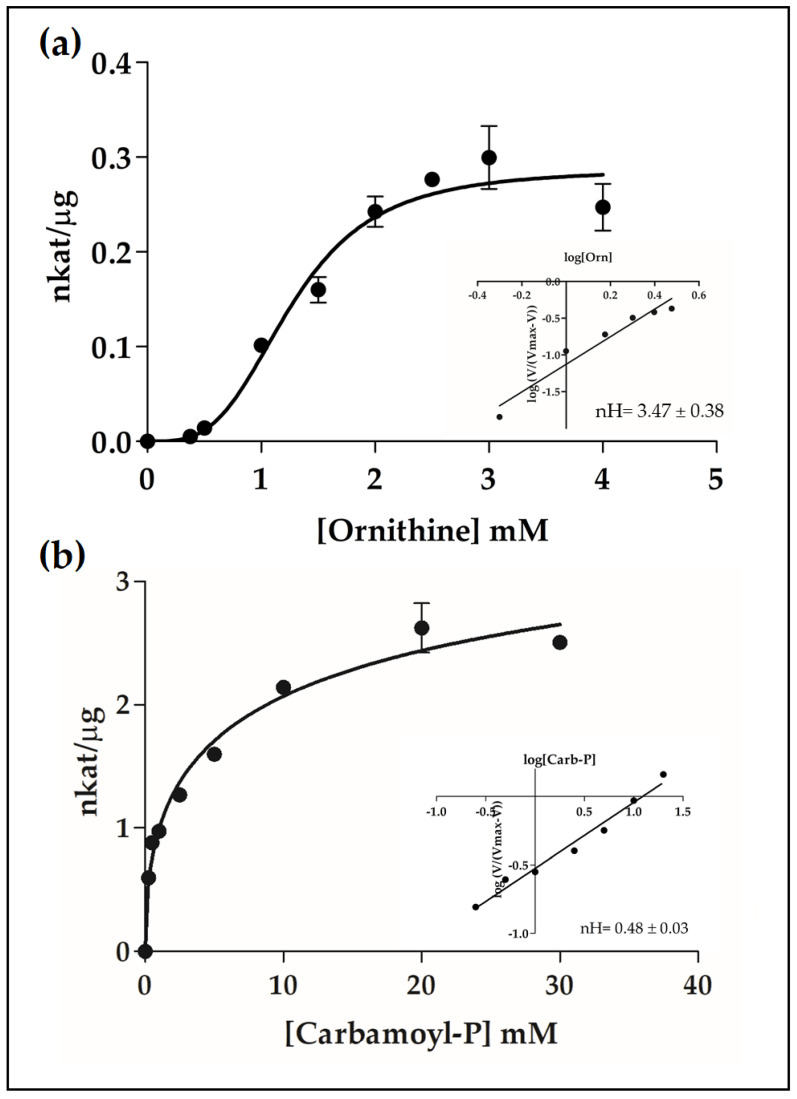
Sigmoidal kinetics of PpOTC. The graph shows the saturation curve of the enzyme in the presence of ornithine (**a**) and carbamoyl-P (**b**). A Hill plot of the kinetic data is shown in the inset (nH = 3.47 ± 0.38 for ornithine; nH = 0.48 ± 0.03 for carbamoyl-P).

To obtain more information about these enzymes and their functional roles, we performed a detailed kinetic analysis using highly purified recombinant enzyme aliquots (Table 1). For PpOTC, our results demonstrated a strong positive cooperativity for ornithine with an estimated nH = 3.5, according to the Hill plot (Figure 4a), and the S_0.5_ value was estimated to be 1.28 mM. On the other hand, our analysis showed negative cooperativity of PpOTC assayed with increasing concentrations of carbamoyl-P, as determined by the Hill plot (nH = 0.48) with a S_0.5_ value of 3.48 mM (Figure 4b). The affinity of the PpASSY towards citrulline and aspartate was 0.12 mM in both cases. The V_max_ for this enzyme was estimated to be 0.06–0.08 nkat/µg, noticeably lower than that of observed for PpOTC or PpASL (Table 1). Finally, the kinetic dynamics of PpASL with various concentrations of argininosuccinate, the single substrate of the reaction, was studied, and Michaelian kinetics was observed with a K_m_ value of 0.22 mM and a V_max_ of 21 nkat/µg.

Based on the determination of the number of reaction centers and kinetic measurements, we estimated the kcat values for the PpOTC, PpASSY and PpASL enzymes. Recombinant PpOTC, with the molecular size of each monomer equal to 38.46 kDa, exhibited a kcat value of 11.03 s^−1^. Recombinant PpASSY, with the molecular size of each monomer equal to 47.44, had a kcat value of 2.82 s^−1^, and a kcat/Km value of 2.46 × 10^4^ M^−1^s^−1^. Finally, recombinant PpASL, with a molecular size of 54.45 kDa for each monomer, showed a kcat value of 1139.24 s^−1^, and a kcat/Km value of 5.18 × 10^6^ M^−1^s^−1^.

### 2.5. Effect of Several Metabolites on PpOTC, PpASSY and PpASL Activities

**Figure 5:**
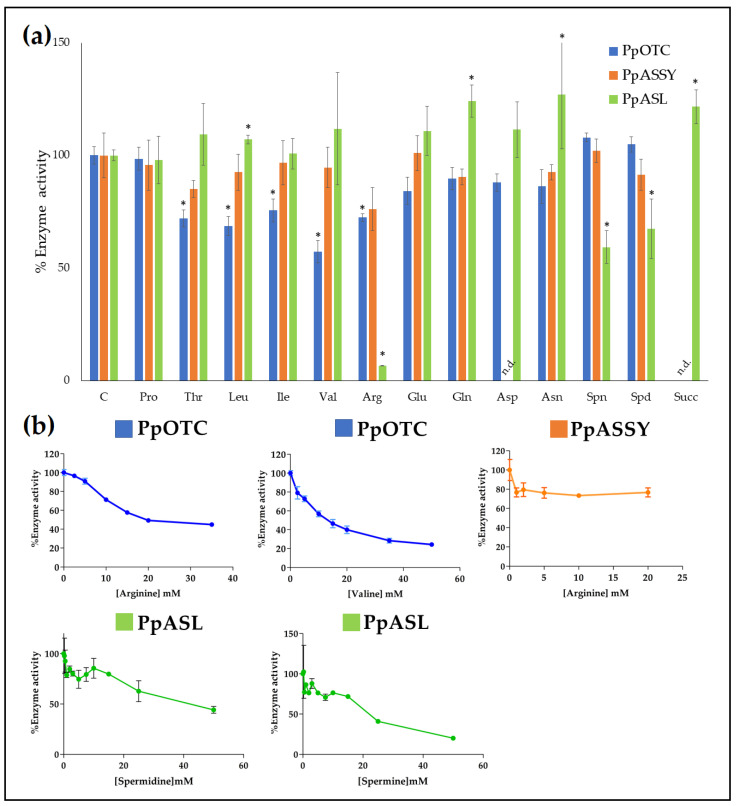
Effect of different compounds on the enzyme activities of PpOTC, PpASSY and PpASL. (**a**) The histogram shows the enzymatic activity of the three enzymes in the presence of different amino acids, spermine (Spn), spermidine (Spd) and succinic acid (Succ). N.d. for no determined data. The enzyme assays were performed in the presence of different compounds at a final concentration of 10 mM as described in the Material and Methods section. Enzyme activity levels in the absence of compounds were considered the controls, C (100%), and the corresponding values were 2.3 ± 0.1 nkat/μg for PpOTC, 0.21 ± 0.02 nkat/μg for PpASSY and 15.63 ± 0.41 nkat/μg for ASL. (**b**) Inhibition plots showing PpOTC, PpASSY and PpASL activities in the presence of increasing amounts of selected effectors indicated in panel (**a**)**.** (*) Significant difference vs. the control. All reactions were carried out with substrate concentrations greater than their respective K_m_ (mM) values at 25 °C with vigorous shaking during 5 min (end point).

Next, a series of enzymatic measurements were performed to determine the putative influence of several metabolites on the metabolic flux from ornithine to arginine. As illustrated in Figure 5a, the activities of PpOTC, PpASSY and PpASL enzymes were determined at saturating concentrations of the corresponding substrates and in combination with selected metabolites at 10 mM, as described in the Materials and Methods section. Enzyme activity in the absence of additional compounds was used as a control (100%). A significant effect (25–40% reduction) on PpOTC activity was observed by the addition of Thr, Leu, Val, and the final product of the pathway, Arg. Interestingly, we also observed a significant slight increase in PpOTC activity, of approximately 10%, by the presence of two polyamines synthesized from arginine: spermine and spermidine. An in-depth analysis of the inhibitory dynamics resulting from Val and Arg addition was performed, and the results showed that concentrations of Arg and Val greater than 20 mM caused a reduction in PpOTC activity of 50% and 70%, respectively (Figure 5b).

**Figure 6:**
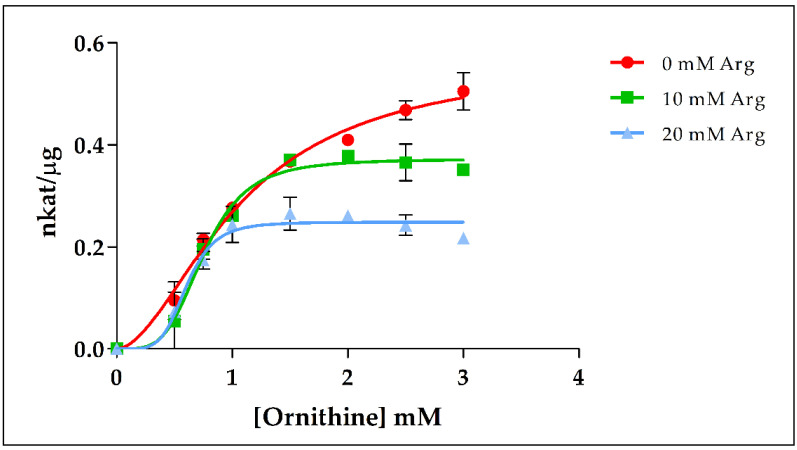
Allosteric inhibition of PpOTC by arginine. The figure shows how the presence of arginine modifies the PpOTC activity, reducing V_max_ but increasing the affinity of the enzyme for ornithine. Values: 0 mM Arg: S_0.5_ = 1.12 ± 0.23 mM, V_max_ = 0.57 ± 0.05 nkat/μg; 10 mM Arg: S_0.5_ = 0.32 ± 0.08 mM, V_max_ = 0.37 ± 0.01 nkat/μg; 20 mM Arg: S_0.5_ = 0.08 ± 0.06 mM, V_max_ = 0.25 ± 0.01 nkat/μg.

Since PpOTC shows strong positive cooperativity with ornithine, the inhibitory effect of Arg on PpOTC activity was also investigated using different concentrations of both Arg and ornithine. As shown in Figure 6, V_max_ was reduced when Arg was increased, with a V_max_ in the presence of 20 mM Arg being one-half that in the absence of this compound, thus indicating a critical role of Arg as a negative allosteric modulator of PpOTC activity.

Next, we investigated the effect of the same set of metabolites on PpASSY activity and determined that only the addition of 10 mM of Arg significantly affected its activity, by approximately 20%. Surprisingly, a detailed study of this effect showed that a similar impact on the PpASSY activity was achieved by Arg concentrations from 1 mM to concentrations greater than 20 mM. Finally, the modulatory impact of these metabolites on PpASL activity was proven. Interestingly, 10 mM of Gln, Glu, Val, Asn, Asp, Leu and Thr resulted in an increase in PpASL activity between 10% and 30%. Previous work reported an inhibitory effect of succinate versus fumarate on the reverse reaction of bovine ASL activity [34] but no effect versus argininosuccinate, as shown here. Interestingly, the presence of succinate increases PpASL activity. In the particular case of PpASL, the presence of polyamines resulted in PpASL activity reduction of approximately 40% and 30% when spermine and spermidine were present, respectively. IC50 values for the different compounds tested are shown in Table 2.

## 3. Discussion

### 3.1. Enzyme Structure and Recombinant Production

Advances in NGS have allowed the establishment of a large body of genomic resources in conifers with multiple applications in fundamental biology and biotechnology. In this study, the genomic resources available for maritime pine were used to explore the function of enzymes involved in the biosynthesis of arginine from ornithine [21] The primary structure of these enzymes is very similar to the corresponding sequences of their counterparts in mammals. Essential protein motifs for the binding of substrates are highly conserved, with only small changes in their amino acid sequences (Figure 2). The most relevant difference among plant and animal polypeptides is the presence of an N-terminal presequence for plastid targeting.

Fully active recombinant enzymes involved in the conversion of ornithine to arginine have not been previously studied in plants. In this work, active OTC, ASSY and ASL enzymes were successfully overexpressed (Figures S2 and S3). In fact, previous attempts at producing recombinant plant enzymes were unfruitful. The Arabidopsis OTC was poorly expressed in *E. coli* and did not complement an auxotrophic mutant [35]. Highly purified preparations were used to estimate their native molecular masses, which were consistent with a homotrimer for PpOTC and a homotetramer for both PpASSY and PpASL holoenzymes in maritime pine (Figure 3). These results are in line with the molecular masses reported for enzymes purified from other organisms [15,36,37].

### 3.2. Enzyme Kinetics

Overproduction of maritime pine enzymes in *E. coli* provided sufficient amounts for the determination of their kinetic and regulatory properties. The affinity of pine OTC, ASSY and ASL for their substrates was in the range of those previously characterized for the counterparts in animals and plants (Table S1): (0.20–3.0 mM) for ornithine and (0.01–1 mM) for carbamoyl-P in OTC; (0.04–0.12) for citrulline and (0.020–0.12) for aspartate in ASSY; (0.02–0.15 mM) for argininosuccinate in ASL. Interestingly, maritime pine OTC showed a sigmoidal behavior against ornithine, suggesting regulation of this enzyme in response to fluctuations of ornithine concentration in the plastid, with low levels of OTC activity when ornithine availability is low and a rapid increase in activity the levels of this substrate are high. It is also remarkable that the negative cooperativity of OTC against carbamoyl-P serves the enzyme to buffer changes in its availability. This kinetic behavior will provide a constant flux of ornithine independently of changes in the concentration of carbamoyl-P in the plastid. As the pathways for arginine and pyrimidine biosynthesis are both located in the plastid, these results strongly suggest that the levels of OTC activity in pine is mainly regulated by the magnitude of the ornithine pool inside the organelle. In this context, it is crucial to consider the activity of arginine and ornithine transporters located in the chloroplast inner membrane. Mitochondrial membrane transporters (BAC1 and BAC2) mediating the translocation of basic amino acids such as arginine, ornithine and lysine have been characterized in *Arabidopsis* [38,39] and are potentially involved in the catabolism of arginine [5]. Unfortunately, very little is known about membrane transporters similarly involved in the interchange of ornithine and arginine between the chloroplasts and the cytosol.

### 3.3. Relevance of the Ornithine-Arginine Conversion in N Metabolism

It has been reported that *Arabidopsis* chloroplasts are able to dissimilate arginine, releasing ornithine and ammonium [40]. This ability can allow a rapid utilization of the N contained in the guanidinium group of this N-rich amino acid because the released ammonium is efficiently assimilated by the GS2-GOGAT cycle in angiosperms. In fact, a citrulline-arginine shuttle was proposed for the metabolic transport of photorespiratory ammonium from mitochondria to chloroplast [40]. As arginine plays an important role in pine as a transient N storage metabolite, the operability of such a mechanism for the rapid interconversion of arginine to ornithine would facilitate a rapid N storage and mobilization. However, no plastidic GS isoform is present in pines and other conifers [41,42], and, therefore, arginine dissimilatory activities would be present in the cytosol in these plants where it will possibly contribute to the cytosolic pool of ornithine. In addition, ornithine is synthesized in the cytosol of plant cells by the action of N-acetyl ornithine deacetylase (NAOD) and serves as a precursor for the biosynthesis of polyamines and alkaloids in this subcellular compartment [4,43]. Thus, ornithine plays a central role as an intermediate metabolite in the crossroads of several pathways. Ornithine is also a precursor of proline and it has been proposed to play a role in the response of plants to abiotic stresses such as salinity and drought [44,45]. Ornithine released by arginase activity during the mobilization of seed storage proteins during germination can be used for glutamate biosynthesis when photosynthetic glutamate synthases are inactive [46].

### 3.4. Effector-Mediated Regulation of Ornithine and Arginine Biosynthesis 

NAGS, the first enzyme in the arginine biosynthetic pathway is allosterically controlled by arginine in prokaryotes and likely in plants [44]. However, the best characterized regulatory step in the arginine biosynthetic pathway is the reaction catalyzed by N-acetylglutamate kinase (NAGK), which is inhibited allosterically by arginine, the end product of the pathway [47]. NAGK is further regulated by the PII protein, which is able to relieve arginine inhibition, allowing the storage of N as arginine [48,49,50]. In plants, when N availability is high, increased levels of glutamine act as a signal that is sensed by PII and transduced in enhanced NAGK activity [51] causing increased flux through the pathway. In maritime pine, two molecular variants of PII, PpPIIa and PpIIb, are able to enhance NAGK activity in the presence of 10 mM of glutamine, with PpPIIa being the isoprotein predominantly involved in the regulation of arginine biosynthesis during embryogenesis [52]. The allosteric regulation of PpOTC by ornithine and arginine (Figure 4 and Figure 6) strongly suggest that the reaction catalyzed by this enzyme is also an important step for the short-term regulation of the pathway. PpOTC activity is also sensitive to the inhibition by Ile, Leu and Val (Figure 5) (reported for animal extracts in [53]) and that suggests a metabolic connection between the aspartate and arginine metabolic pathways as previously pointed out for the biosynthesis of amino acids [54]. The observed inhibition of the last enzyme in arginine biosynthesis, ASL, by spermine and spermidine may reflect the fine-tuned control that polyamines, the arginine and ornithine byproducts, may exert over the biosynthesis of their own precursors.

In summary, our studies address a highly regulated pathway with several points of control allowing the use of ornithine as a precursor placed at the crossroads of several metabolic pathways in different subcellular compartments. The results presented in this study provide a molecular basis for the regulation of ornithine and arginine metabolism in plants at the enzymatic level. The reaction catalyzed by OTC emerges as a key regulatory point controlling the balance between subcellular ornithine pools that can be either used for the biosynthesis of arginine in the plastid and other nitrogenous compounds in the cytosol. Consistently, it is not surprising that ornithine biosynthesis and utilization are the targets for the action of pathogen toxins such as phaseolotoxin and mangotoxin [55,56]. As a product of arginase, ornithine is also a metabolic intermediary for arginine catabolism in the mitochondria. The activity of plastidic and mitochondrial transporters for arginine and ornithine likely play essential roles in maintaining N homeostasis during maritime pine embryogenesis and germination. The functional analysis of these membrane proteins deserves further attention in future studies.

## 4. Material and Methods

### 4.1. RNA Isolation and cDNA Cloning

Maritime pine seeds (Sierra de Segura) were submerged in water with constant aeration for 24 h. Soaked seeds were spread between two layers of vermiculite (Vermiculita Nº3, Projar S.A.) for 30 days, adding only distilled water for seed germination and seedling growth. Cotyledons of seedlings were harvested and quickly frozen in liquid nitrogen. Frozen cotyledons were pulverized, and total RNA was extracted and isolated following the protocol described by de la Torre et al. [57]. RNA was converted to cDNA using iScript reversotranscriptase (BioRad) as previously described [58].

Oligonucleotides for the corresponding ORF cloning were designed by retrieving maritime pine OTC, ASSY and ASL (PpOTC, PpASSY and PpASL) sequences from maritime pine data available at Congenie database [21]. Oligonucleotides were designed avoiding the sequence corresponding to the chloroplast transit peptide sequence (Table S1).

Cloned cDNAs were 984 nucleotides in length for PpOTC, 1218 nucleotides for PpASSY and 1398 nucleotides for PpASL. attB1 and attB2 ends were added by PCR using specific primers (Table S1), necessary for Gateway^®^ cloning (Invitrogen™, Thermo Fisher Scientific™). Sequences were inserted into the pDONR207 vector via Gateway^®^ BP II clonase mix (Invitrogen™, Thermo Fisher Scientific™) reaction and later inserted into the pDEST17 expression vector via Gateway^®^ LR clonase II mix (Invitrogen™, Thermo Fisher Scientific™) reaction following manufacturer’s indications. pDEST17 with inserted cloned sequences were sequenced to confirm correct insertion of cDNA sequence.

### 4.2. Overexpression of Recombinant Enzymes

*Escherichia coli* BL21 AI (Thermo Fisher Scientific™), RIL (BL21 Codon Plus DE3 RIL ™, Thermo Fisher Scientific™) and LEMO (BL21 Codon Plus DE3 Lemo System ™, New England BioLabs^®^) strains were transformed with the pDEST17 plasmid for recombinant protein production. Different growth and production conditions were tested until reaching the optimals or overproduction of each recombinant protein (Table 1). Transformed cells were grown at 37 °C temperature with shaking in Luria Bertani broth containing 100 μg mL^−1^ ampicillin and 25 μg mL^−1^ chloramphenicol until OD_600_ = 0.5 was reached. Induction of protein production was performed by addition of 0.4 mM IPTG (Isopropyl-β-D-thiogalactoside #sc-202185B SantaCruz Biotechnology) for PpOTC and 0.5 mM IPTG for PpASSY. No IPTG was required for PpASL production. Temperature was set to 30 °C for induction of PpOTC, 25 °C for PpASSY and 37 °C for PpASL. Cultures were maintained under their optimal temperature and gentle shaking (60–75 rpm) for 8 h (PpOTC) and 5 h (PpASSY and PpASL). Bacterial cultures were centrifuged at 5000× *g* and pellets were frozen. Proteins were purified by HisTag affinity onto Ni-agarose resin (Protino™ NiTED HisTag purification kit; Macherey-Nagel^®^). Aliquots from the different purification steps were analyzed by SDS-PAGE to follow the progress of purification (Figure S1). Protein concentration was determined via western blot using a known concentration HisTag recombinant protein as reference. The antibodies used were anti-His 6 (Roche Life Science) and anti-mouse HRP-conjugate (Santa Cruz Biotechnology) at dilutions at 1:5000 and 1:10,000, respectively. SDS-PAGE and western blot analysis were carried out as described previously [59,60].

The purified preparations of recombinant enzymes were loaded onto Sephadex G-25 in PD-10 desalting columns to be separated from small molecules and ions that could interfere during enzymatic assays. Collected aliquots were utilized to perform the assays.

### 4.3. Enzyme Assays

The enzyme OTC catalyzes the following reaction:

Ornithine + Carbamoyl-P → Citrulline + Pi

PpOTC activity was determined by quantification of the Pi released with molybdate, that turns into the colored compound phosphomolybdate. The protocol used is the one described by Bernhart and Wreath [61], with minor modifications. Briefly, the reaction mixture in a total volume of 100 μL of 50 mM Tris-HCl (pH 8.0), 1 mM L-ornithine, 5 mM carbamoyl-P and 1.2 μg of the purified recombinant protein was incubated at 25°C for a range of time from 1 to 5 min. The reaction was stopped by the addition of 800 μL of a molybdate solution containing 50% (*v*/*v*) acetone, 5 N H_2_SO_4_ and 5% (*w*/*v*) ammonium molybdate in proportions (2:1:1)). Absorbance was measured at 430 nm, 12 s after the arrest of the reaction. The concentrations used ranged from: 375 μM to 4 mM, for ornithine and between 250 μM and 30 mM for carbamoyl-P. Pi contents were calculated based on a standard curve using commercial sodium phosphate (Merck, Darmstadt, Germany).

The ASSY enzyme catalyzes the following reaction:

Citrulline + Aspartate + ATP → Argininosuccinate + AMP + PPi

PpASSY activity was determined by measuring of the released PPi and its reaction with molybdate, forming 18-molybdopyrophosphate. This compound turns blue when reduced in presence of ascorbic acid and has a peak of absorbance at 790 nm, as described previously by Katano H. et al. [62]. The reaction mixture contained in a total volume of 100 μL 50 mM Tris-HCl (pH 8), 2 mM citrulline, 2 mM aspartate, 3 mM adenosin triphosphate (ATP), 10 mM MgCl_2_ and 2.4 μg of purified PpASSY. Incubation was performed at 25 °C for a range of times from 1 to 8 min. Aliquots of 10 μL were taken each minute and the enzyme reaction stopped by adding 10 μL of an EDTA-NaOH solution containing 400 mM EDTA, 1.4 M NaOH. A volume of 200 μL of a molybdate solution containing 60% (*v*/*v*) acetonitrile, 5% (*v*/*v*) 12 N HCl and 20 mM sodium molybdate was added, and the resulting solutions were incubated at room temperature (RT) in darkness for 10 min. A volume of 80 μL of a solution containing 60% (*v*/*v*) acetonitrile, 5% (*v*/*v*) 12 N HCl and 500 mM ascorbic acid (#A5960-100G, Sigma-Aldrich) was added to a final volume of 300 μL, incubated at room temperature for 10 min and absorbance read at 790 nm. The concentrations used ranged from 50 μM to 2 mM for both citrulline and aspartate. PPi contents were calculated based on a standard curve using commercial sodium pyrophosphate (Merck, Germany).

The ASL catalyzes the reaction:

Argininosuccinate → arginine + fumarate

PpASL activity was determined by measuring absorbance of fumarate at 240 nm. The reaction mixture contained in a volume of 100 μL 50 mM Tris-HCl (pH 8), 2 mM argininosuccinate and 13.2 μg of the purified PpASL. The reaction was incubated at 25 °C for 8 min, measuring the absorbance each 30 s. The concentrations used ranged from 25 μM to 2 mM of argininosuccinate. Fumarate contents were calculated based on a standard curve using commercial fumaric acid (Merck, Germany). 

For the three enzymes, apparent K_m_ and V_max_ values were determined using PRISM5 software (nonlinear analysis: “allosteric sigmoidal” for ornithine and carbamoyl-P and “Michaelis-Menten” for citrulline, aspartate and argininosuccinate) (GraphPad Software LLC).

### 4.4. Size-Exclusion Chromatography

Desalted aliquots of purified recombinant enzymes (PpOTC, PpASSY and PpASL) were loaded separately onto a size-exclusion ENrich™ SEC 650 column (BioRad) in order to determine the size of the multimer. The column was calibrated using commercial protein standards: thyroglobulin (669 kDa), apoferritin (443 kDa), *β*-amylase (200 kDa), alcohol dehydrogenase (150 kDa) and carbonic anhydrase (29 kDa) (Gel Filtration Markers Kit MWGF1000 from Sigma-Aldrich, MO, USA). The fractions were collected, and the elution of the holoenzymes was determined by measuring absorbance at 280 nm and further confirmed by western blot analysis [58].

## Figures and Tables

**Table 1:** Kinetic parameters for recombinant PpOTC, PpASSY and PpASL.

Enzyme	Substrate	K_m_ (mM)	S_0.5_ (mM)	V_max_ (nkat/μg)	nH	k_cat_ (s^−1^)	k_cat_/K_m_ (M^−1^s^−1^)
PpOTC	ornithine		1.28 ± 0.16	4.44 ± 1.4	3.47 ± 0.38	11.03	
	carbamoyl-P		3.48 ± 1.4		0.48 ± 0.03		
PpASSY	citrulline	0.12 ± 0.03		0.07 ± 0.01		2.82	2.46 × 10^4^
	aspartate	0.12 ± 0.05					
PpASL	argininosuccinate	0.22 ± 0.03		20.93 ± 0.75		1139.24	5.18 × 10^6^

**Table 2:** IC50 values for PpOTC, PpASSY and PpASL activity inhibitor compounds.

Enzyme	Compound	IC50 Value (mM)
PpOTC	arginine	10.28
	valine	7.19
PpASSY	arginine	1.94
PpASL	spermidine	4.97
	spermine	19.24

## Supplementary Materials

The following files are available online at https://www.mdpi.com/2223-7747/9/10/1271/s1, Figure S1: Alignment of OTC, ASSY and ASL polypeptides from different species; Figure S2: Purification progress of recombinant maritime pine enzymes; Figure S3, Kinetics plots for the substrates of PpOTC, PpASSy and PpASL; Table S1: List of Primers used for cDNA cloning PpOTC, PpASSY and PpASL from *Pinus pinaster* cDNA and Gateway^®^ BP reaction.

## References

[1] Ågren G.I., Wetterstedt J.Å.M., Billberg M.F.K. (2012). Nutrient limitation on terrestrial plant growth—Modeling the interaction between nitrogen and phosphorus. New Phytol..

[2] Cañas R.A., de la Torre F., Pascual M.B., Ávila C., Cánovas F.M. (2016). Nitrogen economy and nitrogen environmental interactions in conifers. Agronomy.

[3] Li G., Coleman G.D. (2019). Nitrogen storage and cycling in trees. Adv. Bot. Res..

[4] Llebrés M.T., Pascual M.B., Debille S., Trontin J.-F., Harvengt L., Ávila C., Cánovas F.M. (2018). The role of arginine metabolic pathway during embryogenesis and germination in maritime pine (*Pinus pinaster* Ait.). Tree Phys..

[5] Winter G., Todd C.D., Trovato M., Forlani G., Funck D. (2015). Physiological implications of arginine metabolism in plants. Front. Plant Sci..

[6] Corpas F.J., Palma J.M., del Río L.A., Barroso J.B. (2009). Evidence supporting the existence of L-arginine-dependent nitric oxide synthase activity in plants. New Phytol..

[7] Krebs H.A., Henseleit K. (1932). Untersuchungen über die Harnstoffbildung im Tierkörper. Klin. Wochenschr..

[8] Mathews C.K., van Holde K.E., Appling D.R., Anthony-Cahill S.J. (2013). Biochemistry.

[9] Cohen S.N., Kuda A. (1996). Argininosuccinate Synthetase and Argininosuccinate Lyase Are Localized Around Mitochondria: An Immunocytochemical Study. J. Cell Biochem..

[10] Summar M.L., Koelker S., Freedenberg D., Le Mons C., Haberle J., Lee H.-S., Kirmse B. (2013). The incidence of urea cycle disorders. Mol. Gen. Metab..

[11] Szefel J., Danilelak A., Kruszewski W.J. (2019). Metabolic pathways of L-arginine and therapeutic consequences in tumors. Adv. Med. Sci..

[12] Durzan D.J. (2009). Arginine, scurvy and Cartiers’s “tree of life”. J. Ethnobiol. Ethnomed.

[13] Shargool P.D., Jain J.C., McKay G. (1988). Ornithine biosynthesis, and arginine biosynthesis and degradation in plant cells. Phytochemistry.

[14] Arabidopsis Genome Initiative (2000). Analysis of the genome sequence of the flowering plant *Arabidopsis thaliana*. Nature.

[15] Slocum R.D. (2005). Genes, enzymes and regulation of arginine biosynthesis in plants. Plant Phys. Biochem..

[16] Fremont N., Riefler M., Stolz A., Schmülling T. (2013). The Arabidopsis TUMOR PRONE5 gene encodes an acetylornithine aminotransferase required for arginine biosynthesis and root meristem maintenance in blue light. Plant Phys..

[17] Xia J., Yamaji N., Che J., Shen R.F., Ma J.F. (2014). Normal root elongation requires arginine produced by argininosuccinate lyase in rice. Plant J..

[18] Allona I., Collada C., Casado R., Aragoncillo C. (1994). 2S Arginine-rich proteins from *Pinus pinaster* seeds. Tree Phys..

[19] Klimaszewska K., Morency F., Jones-Overton C., Cooke J. (2004). Accumulation pattern and identification of seed storage proteins in zygotic embryos of *Pinus strobus* and in somatic embryos from different maturation treatments. Physiol. Plant.

[20] Trontin J.F., Teyssier C., Morel A., Harvengt L., Lelu-Walter M.-A., Park Y.-S., Bonga J.M., Moon H.-K. (2016). Prospects for new variety deployment through somatic embryogenesis in maritime pine. Vegetative Propagation of Forest Trees.

[21] Cañas R.A., Li Z., Pascual M.B., Castro-Rodríguez V., Ávila C., Sterck L. (2017). The gene expression landscape of pine seedling tissue. Plant J..

[22] Cañas R.A., Pascual M.B., de la Torre F.N., Ávila C., Cánovas F.M. (2019). Resources for conifer functional genomics at the omics era. Adv. Bot. Res..

[23] Shi D., Morizono H., Yu X., Tong L., Allewell N.M., Tuchman M. (2001). Human ornithine transcarbamoylase: Crystallographic insights into substrate recognition and conformational changes. Biochem. J..

[24] Shaheen N., Kobayashi K., Terazono H., Fukushige T., Horiuchi M., Takeyori S. (1994). Characterization of Human Wild-Type and Mutant Argininosuccinate Synthetase Proteins Expressed in Bacterial Cells. Enz. Prot..

[25] Lemke C.T., Howell P.L. (2001). The 1.6A crystal structure of *E. coli* argininosuccinate synthetase suggests a conformational change during catalysis. Structure.

[26] Karlberg T., Collins R., van den Berg S., Hammarström M., Högbom M., Schiavone L.H., Uppenberg J. (2008). Structure of human argininosuccinate synthetase. Acta Cryst. Sect. D.

[27] Sampaleanu L.M., Codding P.W., Lobsanov Y.D., Tsai M., Smith G.D., Horvatin C., Howell P.L. (2004). Structural studies of duck δ2 crystallin mutants provide insight into the role of Thr161 and the 280s loop in catalysis. Biochem. J..

[28] Sampaleanu L.M., Yu B., Howell P.L. (2002). Mutational Analysis of Duck δ2 Crystallin and the Structure of an Inactive Mutant with Bound Substrate Provide Insight into the Enzymatic Mechanism of Argininosuccinate Lyase. J. Biol. Chem..

[29] Lee H.-J., Chiou S.-H., Chang G.-G. (1993). Inactivation of the endogenous argininosuccinate lyase activity of duck δ-crystallin by modification of an essential histidine residue with diethyl pyrocarbonate. Biochem. J..

[30] Bhaumik P., Koski K.M., Bergmann U., Wierenga R.K. (2004). Structure determination and refinement at 2.44 Å resolution of argininosuccinate lyase from *Escherichia coli*. Acta Cryst. Sect. D.

[31] Almagro-Armenteros J.J., Salvatore M., Winther O., Emanuelsson O., von Heijne G., Elofsson A., Nielsen H. (2019). Detecting Sequence Signals in Targeting Peptides Using Deep Learning. Life Sci. All..

[32] Horton P., Park K.-J., Obayashi T., Fujita N., Harada H., Adams-Collier C.J., Nakai K. (2007). WoLF PSORT: Protein localizator predictor. Nucleic Acids Res..

[33] Song Q., Joshi M., DiPiazza J., Joshi V. (2020). Functional Relevance of Citrulline in the Vegetative Tissues of Watermelon During Abiotic Stresses. Front. Plant Sci..

[34] Raushel F.M., Nygaard R. (1983). Kinetic Mechanism of Bovine Liver Argininosuccinate Lyase. Arch. Biochem. Biophys..

[35] Slocum R.D., Nichols III H.F., Williamson C.L. (2000). Purification and characterization of *Arabidopsis* ornithine transcarbamoylase (OTCase), a member of a distinct and evolutionary-conserved group of plant OTCases. Plant Physiol. Biochem..

[36] Kimball M.E., Jacoby L.B. (1980). Purification and properties of argininosuccinate synthetase from normal and canavanine-resistant human lymphoblasts. Biochemistry.

[37] Tuner M.A., Simpson A., McInnes R.R., Howell P.L. (1997). Human argininosuccinate lyase: A structural basis for intragenic complementation. Proc. Natl. Acad. Sci. USA.

[38] Palmieri L., Todd C.D., Arrigoni R., Hoyos M.E., Santoro A., Polacco J.C., Palmieri F. (2006). *Arabidopsis* mitochondria have two basic amino acid transporters with partially overlapping specificities and differential expression in seedling development. Biochim. Biophys. Acta.

[39] Monné M., Miniero D.V., Daddabbo L., Palmieri L., Porcelli V., Palmieri F. (2015). Mitochondrial transporters for ornithine and related amino acids: A review. Amino Acids.

[40] Ludwig R.A. (1993). *Arabidopsis* chloroplasts dissimilate L-arginine and L-citrulline for use as N source. Plant Physiol..

[41] Cánovas F.M., Ávila C., Cantón F.R., Cañas R.A., de la Torre F. (2007). Ammonium assimilation and amino acid metabolism in conifers. J. Exp. Bot..

[42] Miyazawa S.I., Nishiguchi M., Futamura N., Yukawa T., Miyao M., Maruyama T.E., Kawahara T. (2018). Low assimilation efficiency of photorespiratory ammonia in conifer leaves. J. Plant Res..

[43] Molesini B., Mennella G., Martini F., Francese G., Pandolfini T. (2015). Involvement of the putative N-acetylornithine deacetylase from *Arabidopsis thaliana* in flowering and fruit development. Plant Cell Physiol..

[44] Kalamaki S.M., Merkoiropoulos G., Kanellis A.K. (2009). Can ornithine accumulation modulate abiotic stress tolerance in Arabidopsis?. Plant Sign. Behav..

[45] Joshi V., Fernie A.R. (2017). Citrulline metabolism in plants. Amino Acids.

[46] Cañas R.A., Villalobos D.P., Díaz-Moreno S.M., Cánovas F.M., Cantón F.R. (2008). Molecular and Functional Analyses Support a Role of Ornithine-δ-Aminotransferase in the Provision of Glutamate Biosynthesis during Pine Germination. Plant Phys..

[47] Ramón-Maiques S., Marina A., Gil-Ortiz F., Fita F., Rubio V. (2002). Structure of Acetylglutamate Kinase, a Key Enzyme for Arginine Biosynthesis and a Prototype for the Amino Acid Kinase Enzyme Family, during Catalysis. Structure.

[48] Llácer J.L., Contreras A., Forchhammer K., Marco-Marín C., Gil-Ortiz F., Maldonado R., Fita I., Rubio V. (2007). The crystal structure of the complex of PII and acetylglutamate kinase reveals how PII controls the storage of nitrogen as arginine. Proc. Natl. Acad. Sci. USA.

[49] Llácer J.L., Fita I., Rubio V. (2008). Arginine and nitrogen storage. Curr. Opin. Struct. Biol..

[50] Chen D., Shao Q., Yin L., Younis A., Zheng B. (2019). Polyamine Function in Plants: Metabolism, Regulation on Development, and Roles in Abiotic Stress Responses. Front. Plant Sci..

[51] Chellamuthu V.R., Ermilova E., Lapina T., Lüddecke J., Minaeva E., Herrmann C., Hartmann M.D., Forchhammer K. (2014). A widespread glutamine-sensing mechanism on the plant kingdom. Cell.

[52] Llebrés M.T., Pascual M.B., de la Torre F.N., Gómez L., Ávila C., Cánovas F.M. (2020). Structural and functional characteristics of two molecular variants of the nitrogen sensor PII in maritime pine. Front. Plant Sci..

[53] Marshall M., Cohen P.P. (1972). Ornithine Transcarbamylase from *Streptococcus faecalis* and Bovine Liver. (II) Multiple binding sites for carbamoyl-P and L-Norvaline, correlation with steady state kinetics. J. Biol. Chem..

[54] Yang Q., Zhao D., Liu Q. (2020). Connections Between Amino Acid Metabolisms in Plants: Lysine as an Example. Front. Plant Sci..

[55] Tam L.Q., Patil S.S. (1972). Mode of action of the toxin from *Pseudomonas phaseolicola.* II. Mechanism of inhibition of bean ornithine carbamoyl transferase. Plant Phys..

[56] Arrebola E., Cazorla F.M., Durán V.E., Rivera E., Olea F., Codina J.C., Pérez-García A., de Vicente A. (2003). Mangotoxin: A novel antimetabolite toxin produced by *Pseudomonas syringae* inhibiting ornithine/arginine biosynthesis. Phys. Mol. Plant Path..

[57] de la Torre F., El-Azaz J., Ávila C., Cánovas F.M. (2014). Deciphering the Role of Aspartate and Prephenate Aminotransferase Activities in Plastid Nitrogen Metabolism. Plant Phys..

[58] Canales J., Rueda-López M., Craven-Bartle B., Ávila C., Cánovas F.M. (2012). Novel insights into regulation of asparagine synthetase in conifers. Front. Plant Sci..

[59] Cánovas F.M., Cantón F.R., Gallardo F., García-Gutiérrez A., de Vicente A. (1991). Accumulation of glutamine synthetase during early development of maritime pine (*Pinus pinaster*) seedlings. Planta.

[60] Cantón F.R., García-Gutiérrez A., Crespillo R., Cánovas F.M. (1996). High-level expression of *Pinus sylvestris* glutamine synthetase in *Escherichia coli*. Production of polyclonal antibodies against the recombinant protein and expression studies in pine seedlings. FEBS Lett..

[61] Berhart D.N., Wreath A.R. (1955). Colorimetric Determination of Phosphorus by Modified Phosphomolybdate Method. Anal. Chem..

[62] Katano H., Tanaka R., Maruyama C., Hamano Y. (2012). Assay of enzymes forming AMP + PPi by the pyrophosphate determination based on the formation of 18-molybdopyrophosphate. Anal. Biochem..

