# Supplementary material for: Enzymes Involved in the Biosynthesis of Arginine from Ornithine in Maritime Pine (Pinus pinaster Ait.)
Source: Plants (Basel). 2020 Sep 27;9(10):1271. doi: 10.3390/plants9101271 (PMC7601404; doi:10.3390/plants9101271)
Supplement: Supplementary file 1 [file plants-09-01271-s001.zip › plants-946873-supplementary-xml/plants-946873-supplementary-xml.docx]

**Supplementary Materials**


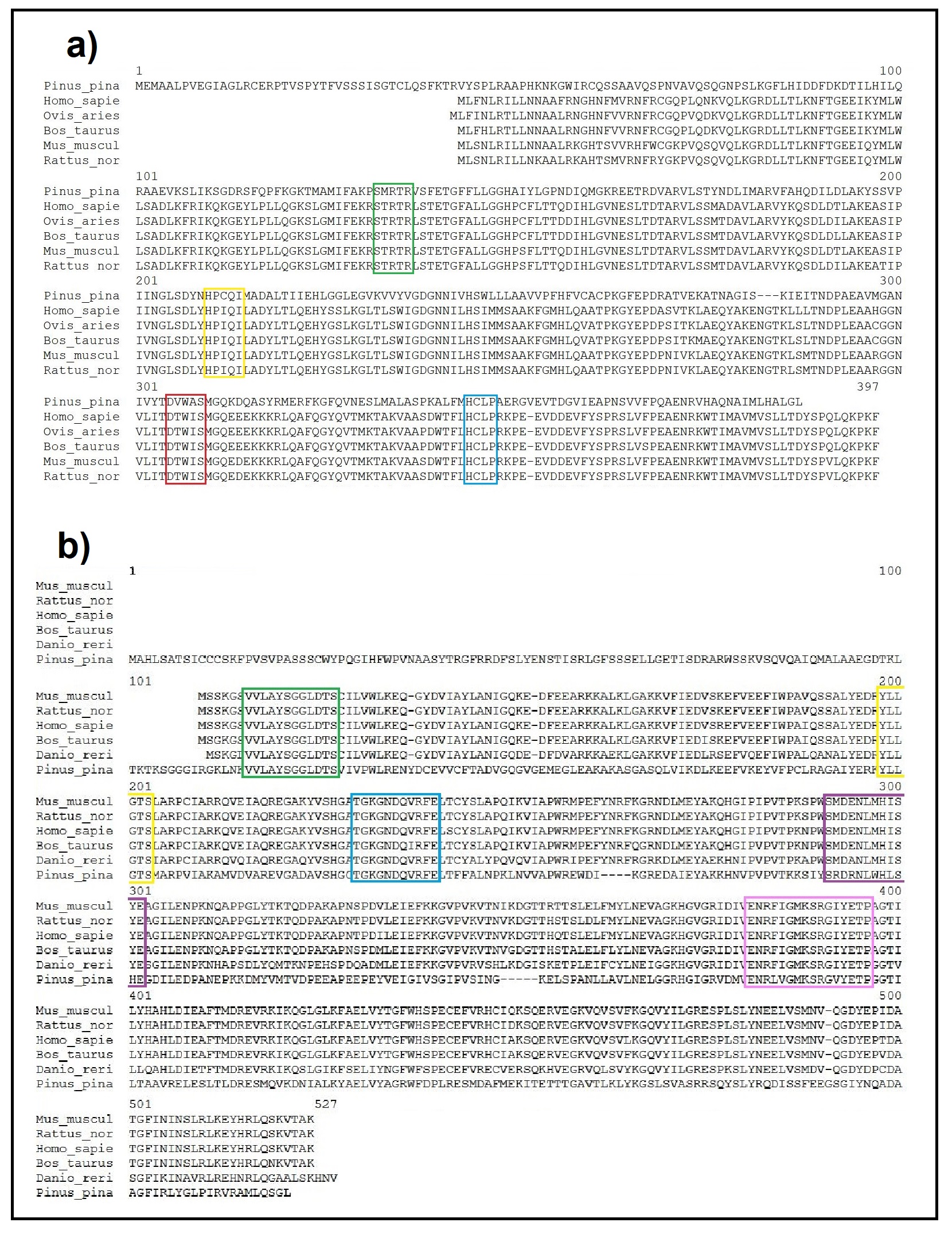


**
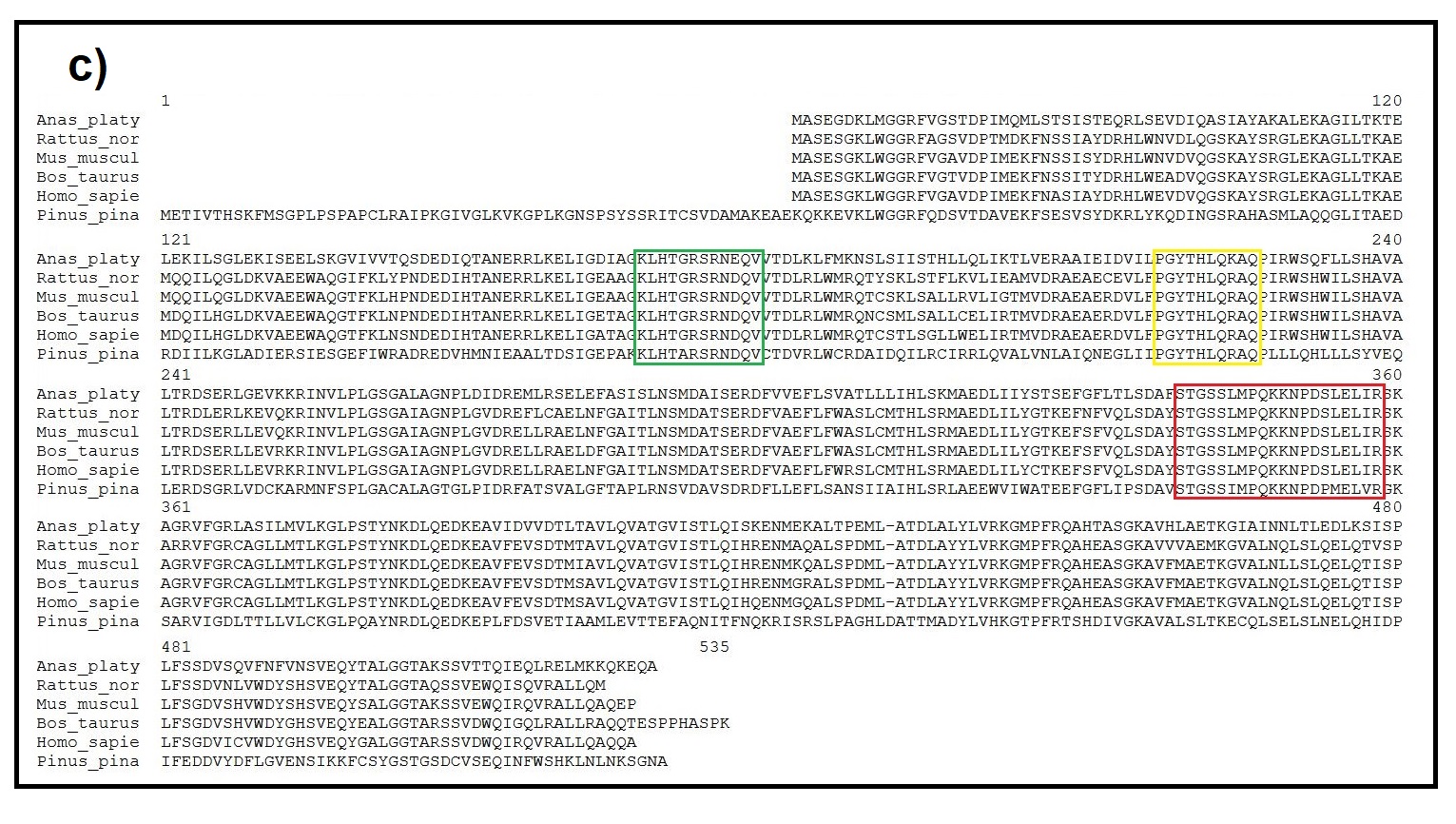
**

**Figure 1.** Alignment of OTC (**a**), ASSY (**b**) and ASL(**c**) polypeptides from different species. Highlighted boxes of the conserved domains for enzyme activity refer to the previously showed on Figure 2.

**Table S1.** Kinetic parameters of OTC, ASSY and ASL (attached file).

**Table S2.** List of Primers used for cDNA cloning PpOTC, PpASSY and PpASL from *Pinus pinaster* cDNA and Gateway ® BP reaction (-attB) (Invitrogen, Thermo Fisher Scientific, USA).

|  | **Forward (5’-3’)** | **Reverse (5’-3’)** |
| --- | --- | --- |
| OTC | ATAAGATGCCAAAGCTCTGCCG | TCAAAGTCCAAGAGCATGCA |
| OTC-attB | AAAAAGCAGGCTTGATAAGATGCCAAAGCTCTGCCGC | AGAAAGCTGGGTTCAAAGTCCAAGAGCATGCA |
| ASSY | GGAATACGTGGAAAATTGAAT | TCATAGTCCACTTTGTAGCATGGC |
| ASSY-attB | AAAAAGCAGGCTTGGGAATACGTGGAAAA | AGAAAGCTGGGTTCATAGTCCACTTTG |
| ASL | AAGGAAGTGAAGCTGTGGGGAG | TCAAGCATTTCCTGATTTGTTTAAA |
| ASL-attB | AAAAAGCAGGCTTGGAAGTGAAGCTGTGG | AGAAAGCTGGGTTCAAGCATTTCCTGA |


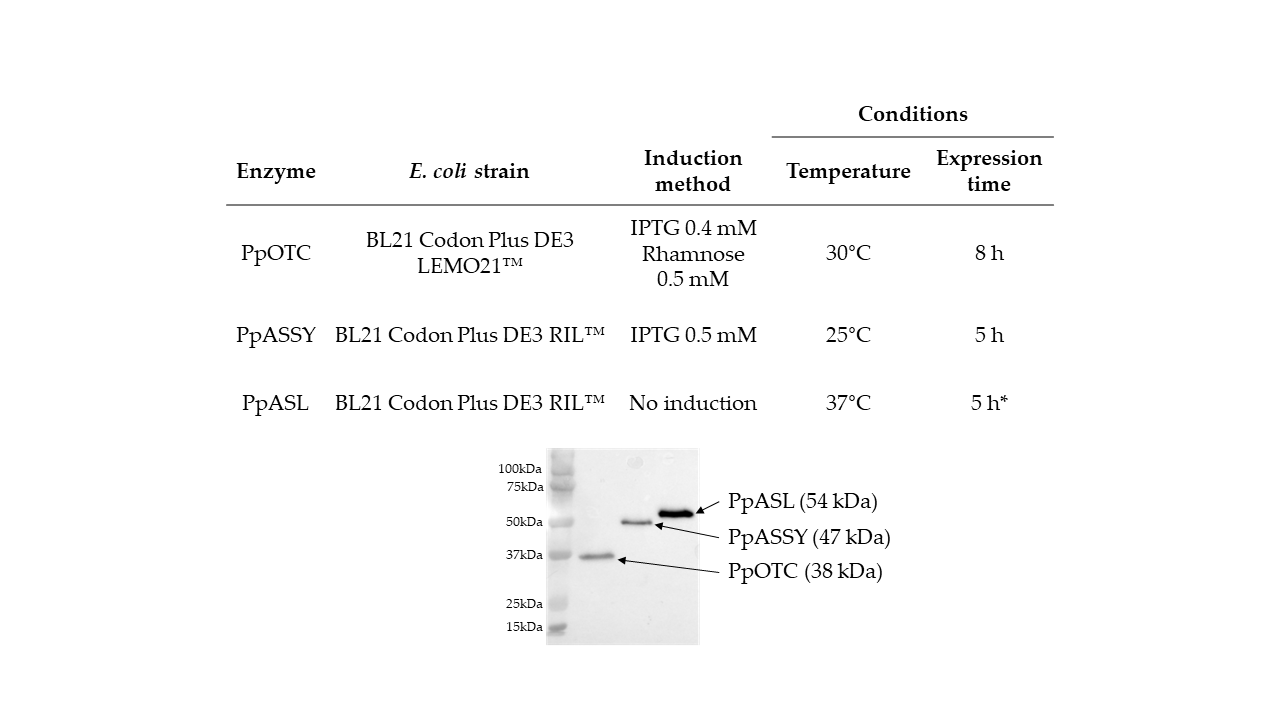


**Figure S2.** Overproduction of recombinant enzymes. After testing different conditions, those finally selected for optimal overproduction of PpOTC (38 kDa), PpASSY (47 kDa) and PpASL (54 kDa) are described. Lemo21™(DE3) (New England Biolabs) and DE3 RIL™ (Thermo Fisher) competent cells were used to overexpress the enzymes. The presence of the maritime pine enzymes in the *E. coli* protein extracts was confirmed by western blot analysis using anti-His antibodies. (*) indicates the time of protein production after reaching OD_600_= 0.5.


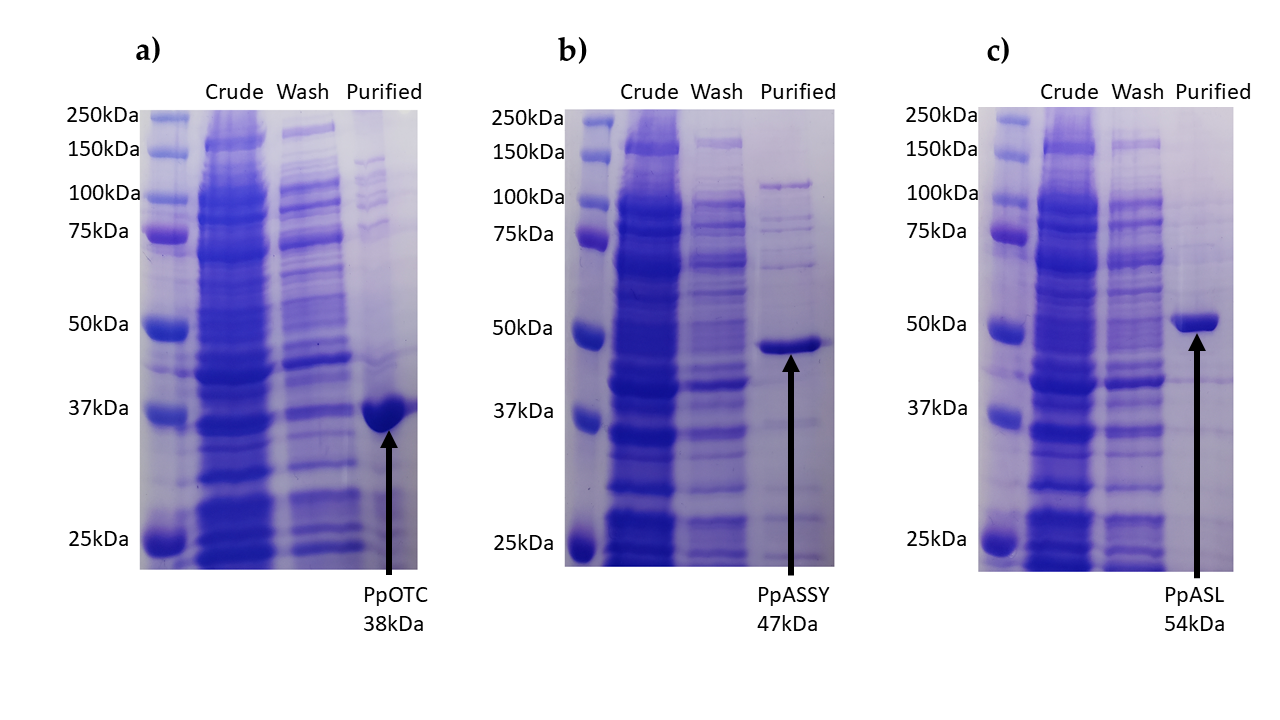


**Figure S3.** Purification progress of recombinant maritime pine enzymes: a) PpOTC, b) PpASSY and c) PpASL. Purified enzymes were used for determination of their molecular properties and kinetic analysis.


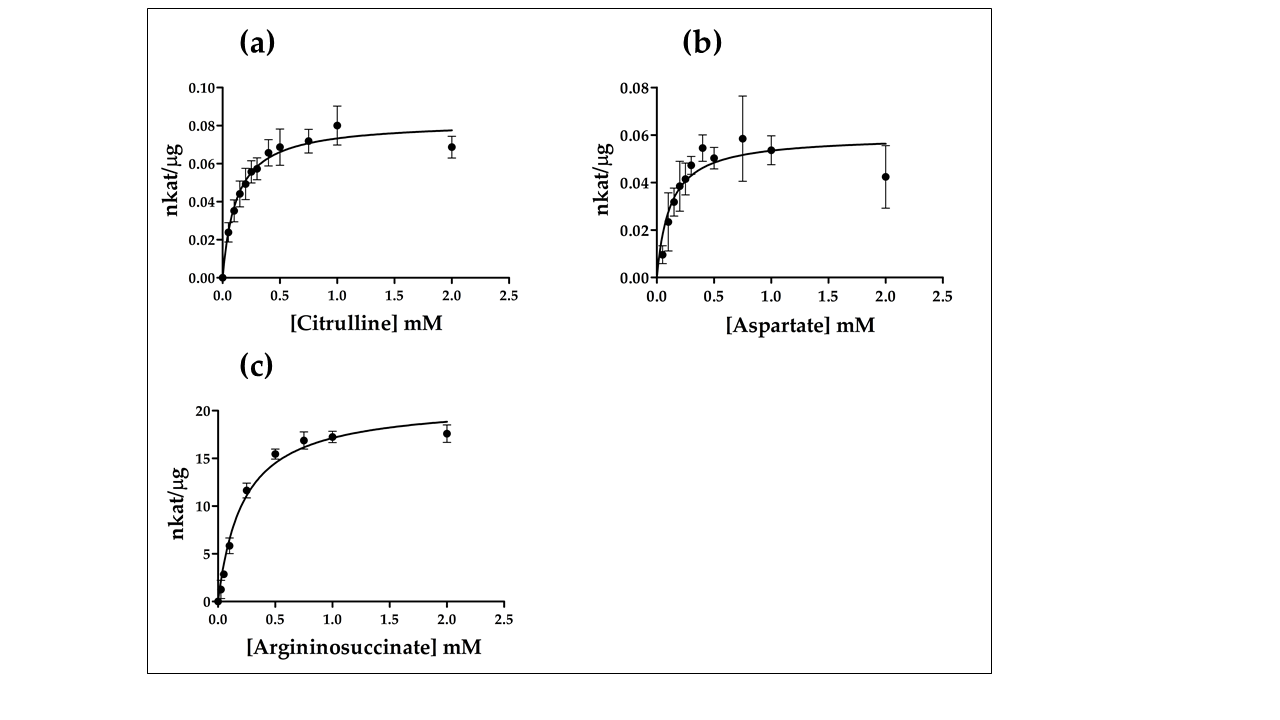


**Figure S4.** Kinetics plots for the substrates of PpOTC (a), PpASSy (b) and PpASL (c).

References for the Supplementary Material

1. Lee, Y.; Choi, Y.-A.; Hwang, I.D.; Kim, S-G.; Kwon, Y.M. cDNA cloning of two isoforms of ornithine carbamoyltransferase from *Canavalia lineata* leaves and the effect of site directed mutagenesis of the carbamoyl phosphate binding site. *Plant Mol. Bio.* **2001**, *46*, 651–660, doi:[10.1023/a:1011632927541](https://doi.org/10.1023/a:1011632927541)
2. Belloco, E.; di Salvo, C.; Lagana, G.; Galtieri, A.; Ficarra, S.; Kotyk, A.; Leuzzi, U. Ornithine carbamoyltransferase from *Spinacea oleracea*: purification and characterization. *Bio. Plant.* **2002**, *45(4)*, 533–538, doi:<https://doi.org/10.1023/A:1022368924239>
3. Glenn, E.; Maretzki, A. Properties and Subcellular Distribution of Two Partially Purified Ornithine Transcarbamoylases in Cell Suspensions of Sugarcane. *Plant Physiol.* **1977**, *60*, 122–126, doi:[10.1104/pp.60.1.122](https://doi.org/10.1104/pp.60.1.122)
4. Tsuji, S. Chicken ornithine transcarbamoylase: purification and some properties *J.* *Biochem.* **1983,** *94*(4), 1307–1315, doi:[10.1093/oxfordjournals.jbchem.a134476](https://doi.org/10.1093/oxfordjournals.jbchem.a134476)
5. Pierson, D.L.; Cox, S.L.; Gilbert, B.E. Human ornithine transcarbamylase. Purification and characterization of the enzyme from normal liver and the liver of a Reye’s syndrome patient. *J. Biol. Chem.* **1977,** *252*(18), [6464–6469](https://www.jbc.org/content/252/18/6464.long).
6. Koger, B.J.; Howell, R.G.; Kelly, M.; Jones, E.E. Purification and properties of porcine liver ornithine transcarbamylase. *Arch. Biochem. Biophys.* **1994,** *309*(2), 293–299, doi:[10.1006/abbi.1994.1116](https://doi.org/10.1006/abbi.1994.1116)
7. Saheki, T.; Kusumi, T.; Takada, S.; Katsunuma, T. Studies of rat liver argininosuccinate synthetase. I. Physicochemical, catalytic, and immunochemical properties. *J. Biochem.* **1977**, *81*(3), 687–696, doi:[10.1093/oxfordjournals.jbchem.a131505](https://doi.org/10.1093/oxfordjournals.jbchem.a131505)
8. Bray, R.C.; Ratner, S. Argininosuccinase form bovine kidney: comparison of catalytic, physical, and chemical properties with the enzyme form bovine liver. *Arch. Biochem. Biophys.* **1971,** *146*(2), 531–541, doi:[10.1016/0003-9861(71)90158-5](https://doi.org/10.1016/0003-9861(71)90158-5)
9. Palekar, A.G.; Mantagos, S. Human liver argininosuccinase purification and partial characterization. *J. Biol. Chem.* **1981,** *256*(17), [9192–9194](https://www.jbc.org/content/256/17/9192.long).
10. Lee, H.-J.; Chiou, S.-H.; Chang, G.-G. Biochemical characterization and kinetic analysis of duck delta crystallin with endogenous argininosuccinate lyase activity. *Biochem* *J*. **1992,** *283*, 597–603, doi:[10.1042/bj2830597](https://dx.doi.org/10.1042%2Fbj2830597)
11. Rosenthal, G.A.; Naylor, A.W. Purification and general properties of argininosuccinate lyase forma jack bean, *Canavalia* *ensiformis* (L.) DC. *Biochem.* *J*. **1969,** *112*(4), 415–419, doi:<https://doi.org/10.1042/bj1120415>
